# Human gnathostomiasis: a neglected food-borne zoonosis

**DOI:** 10.1186/s13071-020-04494-4

**Published:** 2020-12-09

**Authors:** Guo-Hua Liu, Miao-Miao Sun, Hany M. Elsheikha, Yi-Tian Fu, Hiromu Sugiyama, Katsuhiko Ando, Woon-Mok Sohn, Xing-Quan Zhu, Chaoqun Yao

**Affiliations:** 1 Hunan Provincial Key Laboratory of Protein Engineering in Animal Vaccines, College of Veterinary Medicine, Hunan Agricultural University, Changsha, 410128, Hunan People’s Republic of China; 2 State Key Laboratory of Veterinary Etiological Biology, Key Laboratory of Veterinary Parasitology of Gansu Province, Lanzhou Veterinary Research Institute, Chinese Academy of Agricultural Sciences, Lanzhou, 730046 Gansu People’s Republic of China; 3 Faculty of Medicine and Health Sciences, School of Veterinary Medicine and Science, University of Nottingham, Sutton Bonington Campus, Loughborough, LE12 5RD UK; 4 Department of Parasitology, National Institute of Infectious Diseases, Tokyo, 162-8640 Japan; 5 Department of Medical Zoology, Mie University School of Medicine, Mie, 514-8507 Japan; 6 Department of Parasitology and Tropical Medicine, Institute of Health Sciences, Gyeongsang National University College of Medicine, Jinju, 52727 Korea; 7 College of Veterinary Medicine, Shanxi Agricultural University, Taigu, Shanxi 030801 People’s Republic of China; 8 Department of Biomedical Sciences and One Health Center for Zoonoses and Tropical Veterinary Medicine, Ross University School of Veterinary Medicine, P.O. Box 334, Basseterre, St Kitts and Nevis

**Keywords:** *Gnathostoma* spp., Gnathostomiasis, Food-borne zoonosis

## Abstract

**Background:**

Human gnathostomiasis is a food-borne zoonosis. Its etiological agents are the third-stage larvae of *Gnathostoma* spp. Human gnathostomiasis is often reported in developing countries, but it is also an emerging disease in developed countries in non-endemic areas. The recent surge in cases of human gnathostomiasis is mainly due to the increasing consumption of raw freshwater fish, amphibians, and reptiles.

**Methods:**

This article reviews the literature on *Gnathostoma* spp. and the disease that these parasites cause in humans. We review the literature on the life cycle and pathogenesis of these parasites, the clinical features, epidemiology, diagnosis, treatment, control, and new molecular findings on human gnathostomiasis, and social-ecological factors related to the transmission of this disease.

**Conclusions:**

The information presented provides an impetus for studying the parasite biology and host immunity. It is urgently needed to develop a quick and sensitive diagnosis and to develop an effective regimen for the management and control of human gnathostomiasis.
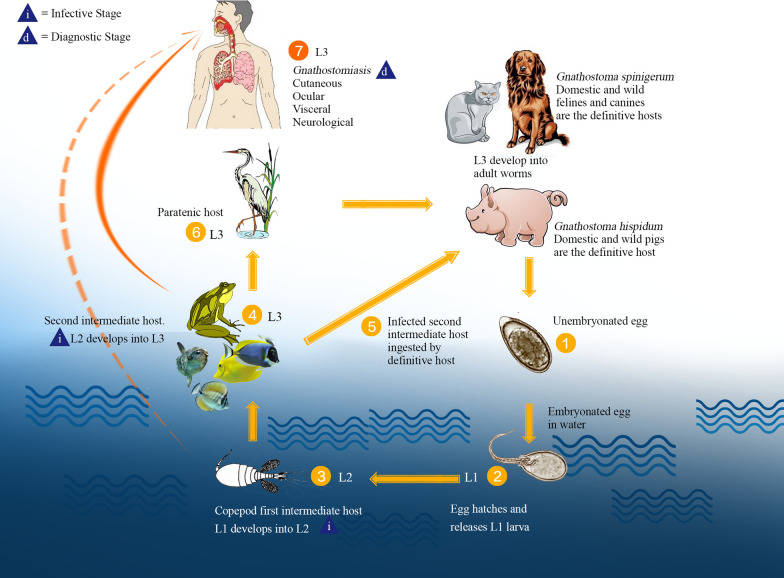

## Background

Human gnathostomiasis, a food-borne zoonosis, is caused by the third-stage larvae (L_3_) of *Gnathostoma* spp. [1]. Humans are infected by these nematodes by consuming raw or undercooked fish, frogs, snakes or poultry that contain the L_3_ [2]. The most common clinical signs and symptoms of the disease are migratory cutaneous swellings and eosinophilia. In severe cases, L_3_ also invade internal organs and tissues such as the liver, eyes, nerves, spinal cord and brain, which can result in blindness, nerve pain, paralysis, coma and even death [3].

The first human case of gnathostomiasis was reported from Thailand in 1889, and was attributed to infection by *Cheiracanthus siamensis* (Levinseen 1889). Shortly afterwards, Leiper (1891) found that *C. siamensis* was morphologically identical to *Gnathostoma spinigerum*, and thus considered the former a synonym of the latter. However, the life cycle of *G. spinigerum* was not elucidated until 1936 [4]. To date, approximately 5000 cases of human gnathostomiasis have been reported worldwide, mainly from endemic areas in Japan and China, Thailand and other parts of Southeast Asia, Mexico, and Colombia and Peru in South America [1, 3]. Gnathostomiasis has also been reported, albeit infrequently, in travelers from developed countries who have visited endemic areas [3, 5–8]. Furthermore, autochthonous gnathostomiasis has been reported in several non-endemic countries [9–12]. Therefore, human gnathostomiasis is considered an emerging global zoonosis [3, 13].

The increase in reports of human gnathostomiasis may be due to changes in eating habits as a result of improved living standards, and also in improvements in health care systems for disease reporting [14]. The eradication of gnathostomiasis is challenging because of the worldwide distribution of *Gnathostoma* spp. and increasing demand for exotic dishes such as marinated or raw fish [1, 14, 15]. Therefore, an effective prevention and control strategy should ideally be implemented for this disease. Here, we comprehensively review several aspects of human gnathostomiasis and discuss future prospects for the improvement of public perception of the importance of this parasitic disease.

## *Gnathostoma* spp. and their life cycles

A gnathostome nematode was first discovered in 1836 in the stomach of a young tiger that had died of aortic rupture at London Zoo [16]. Since then, *Gnathostoma* spp. (Nematoda: Gnathostomatidae) have been determined to be the etiological agents of human gnathostomiasis [17]. Among the 12 species in the genus, at least five, *G. binucleatum*, *G. doloresi*, *G. hispidum*, *G. nipponicum* and *G. spinigerum*, cause human disease [18, 19]. The species most frequently found in humans and most widely distributed around the world is *G. spinigerum*; *G. binucleatum* is found in the Americas [1]. Sporadic cases caused by *G. doloresi*, *G. hispidum*, and *G. nipponicum* have been documented in Asia [20–23].

Gnathostome eggs are oval in shape and have a mucoid plug at one or both ends, depending on the species [24]. The early L_3_ (EL_3_) and the advanced L_3_ (AdL_3_) of *G. spinigerum* in the second intermediate host usually measure 0.85–1.38 in length × 0.10–0.15 mm in diameter and 2.30–4.40 in length × 0.25–0.43 mm in diameter, respectively. AdL_3_ have a characteristic head bulb of 93 × 221 μm on average, which often bears four rows, and occasionally five rows, of hooklets, a long muscular esophagus 0.63–1.22 mm in length and two pairs of cervical sacs 0.33–0.75 mm in length.

**Fig. 1:**
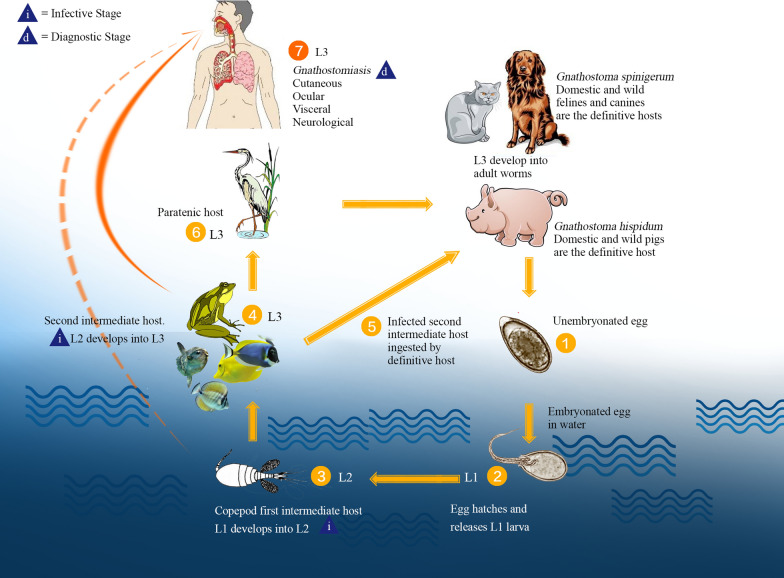
Life cycle of *Gnathostoma*.* L1* First-stage larva, * L2* second-stage larva, * L3* third-stage larva. Adapted from the Centers for Disease Control and Prevention (CDC) DPDx website (https://www.cdc.gov/dpdx/gnathostomiasis/index.html)

*Gnathostoma* nematodes require two intermediate hosts and one definitive host to complete their life cycles (Fig. 1). In general, the adult worms live and spawn in a tumor-like mass in the stomach of the definitive host (e.g., cat, tiger, leopard or dog in the case of *G. spinigerum*). The eggs are released in the host’s feces into the environment, where they develop and hatch into the first-stage larvae (L_1_) in freshwater within 7 days at 28 °C. L_1_ are then ingested by the first intermediate host, freshwater copepods (usually of the genera *Cyclops*, *Eucyclops* and *Mesocyclops*), where they develop into the second-stage larvae (L_2_). When the infected copepods are consumed by the second intermediate host such as a fish or tadpole, L_2_ migrate into the new host’s muscular tissue where they develop into L_3_. If L_3_ in the second intermediate host, transport or paratenic host are ingested by a definitive host (e.g., dogs, cats, pigs or weasel), they migrate to the liver and the abdominal cavity after penetrating the gastric wall. Four weeks later they return to the gastric wall [1] and develop into adults. This development from L3 to adult usually takes 6-8 months. The definitive host starts to excrete the parasite eggs into the environment in its feces approximately 8 to 12 months after ingestion of L_3_ [25, 26]. When L_3_ are eaten by paratenic hosts such as frogs, snakes, birds and mammals including humans, they migrate through their tissues and remain encysted in their muscles.

Human gnathostomiasis can occur through three modes of transmission: oral, transplacental and skin wounds. However, this parasitic disease is mainly caused by the ingestion of raw or undercooked meat of intermediate hosts, such as fish, frogs, snakes or poultry, which contains L_3_ [27]. Oral infection can also occur through drinking water contaminated with infected copepods [28]. Transplacental infection only occurs in pregnant women with a heavy infection of gnathostome larvae, which is rare [29]. L_3_ harbored in the infected meat of intermediate hosts can penetrate the skin of humans, particularly through wounds [30].

## Pathogenesis and clinical presentation

Humans are not definitive hosts of *Gnathostoma* spp., and L_3_ cannot mature into adults in them [31]. L_3_ can, however, cause damage to their tissues and/or organs by inducing host reactions, like inflammation and allergy, when they migrate and secrete excreta and toxins [32]. L_3_ may cause damage to vital organs and the central nervous system (CNS), resulting in detrimental outcomes including the sudden death of an infected individual [33, 34]. The larvae release excretory-secretory products (ES) with divergent functions that contribute to different parasite behaviors including cutaneous and visceral larva migrans [35, 36]. Recent studies have demonstrated that *G. spinigerum* ES antigens modulate monocyte function via inhibition of Fc gamma receptor I expression, and trigger apoptosis of the peripheral blood mononuclear cells mainly via the extrinsic pathway [37, 38].

*Gnathostoma* larvae can migrate to the skin though subcutaneous tissue, and penetrate other tissues and organs including the eyes, ears, breasts, lungs, gastrointestinal tract, thoracic spinal cord, genitourinary system and CNS [8, 39]. Clinical features mostly manifest as cutaneous and visceral migrans, depending on which parts of the body have been invaded. Within 1 or 2 days of ingestion, a *Gnathostoma* larva migrates through the gastrointestinal tract wall and the liver. Patients may develop systemic symptoms and signs such as fever, anorexia, nausea, vomiting, abdominal pain, joint pain etc., which may last for more than 2 weeks. Cutaneous gnathostomiasis involves migrating lumps, which are the most important features in diagnosis of the disease. Clinical manifestations of the various organs infected by *Gnathostoma* spp. differ. A significant increase in eosinophils is common and can be used as a basis for auxiliary diagnosis [40]. The most common form of infection is larval migration within skin tissues, which causes a great amount of pain and lasts for 3–4 weeks. The pathogenesis of gnathostomiasis remains largely unknown. Nevertheless, it is plausible that the symptoms and signs of the disease are due to the mechanical damage caused by larval migration, the inflammation and infection that is secondary to the mechanical damage, the combined effects of reactions to larval ES and the activation of an immune response in the host.

### Cutaneous gnathostomiasis

Cutaneous gnathostomiasis, which is always accompanied by nodular migratory panniculitis [40], is the most common clinical manifestation of human gnathostomiasis. The larvae spread throughout the body (limbs, face, back, abdomen, armpits, breasts etc.) by migrating through the epidermis, dermis and subcutaneous tissue, causing cutaneous larva migrans and resulting in skin irritation, pain and pruritus [41]. Reports of six cases of cutaneous gnathostomiasis noted that it takes an average of 12 days for the condition to be diagnosed and for a patient to start treatment [42, 43]. In these cases, larvae were found in the dermis and subcutaneous tissue by pathological examination of the skin lesions, which were infiltrated with numerous eosinophils along with low numbers of lymphocytes and neutrophils. L_3_ can survive in the human body for a very long period of time; episodes of swelling may become brief and less intense, and symptoms may recur intermittently for more than 10 years in untreated patients. Cutaneous gnathostomiasis should be suspected in a patient with creeping eruption, migratory swellings, a skin abscess or nodules [44].

### Visceral gnathostomiasis

*Gnathostoma* larvae, which are highly invasive, can migrate throughout various internal organs, resulting in a wide range of symptoms and signs that can affect almost any part of the body. In the visceral disease, larvae may cause intermittent symptoms for a long period of time or, without proper treatment, until the host’s death [1].

#### Ocular manifestations

*Gnathostoma* larvae can invade the eyes, leading to external and internal ocular lesions with inflammation, and other symptoms and signs such as subarachnoid hemorrhage, and even permanent vision loss [34, 45]. Clinical manifestations such as occasional eyelid edema, conjunctival pain and conjunctival erythema have also been reported [46]. Larvae in the eyes can be visualized, and are generally found in the anterior chamber. With surgical removal of larvae, visual performance can be recovered completely, but optic neuropathy can occur leading to permanent blindness [47, 48].

#### Auricular manifestations

L_3_ can damage the inner ear leading to tinnitus, dizziness, hearing loss and other symptoms.

#### Pulmonary manifestations

Clinical manifestations in patients with pulmonary involvement include fever, cough, chest pain, nodular densities and pneumothorax, mostly accompanied by complicated pleural effusion [49]. Peripheral blood eosinophils in patients with pulmonary manifestations were found to be significantly increased [29]. Lung cancer patients infected with *Gnathostoma* spp. suffered repeated fevers, cough, chest tightness and other respiratory non-specific symptoms [1].

#### Gastrointestinal manifestations

Gastrointestinal manifestations of gnathostomiasis in humans include sharp abdominal pain, anorexia, vomiting, and indigestion as the larvae invade the stomach wall, which causes a large area of gastric mucosal inflammatory congestion and can result in a gastric ulcer or gastric perforation, and even acute right iliac fossa pain [1].

#### Genitourinary manifestations

Larvae can pass through bladder tissue into the urine, and symptoms of this may include hematuria, the sensation of a foreign body in the urine, etc. Urinary tract disease is rare [50–52].

#### CNS manifestations

Invasion of the CNS by *Gnathostoma* L_3_ causes neurognathostomiasis [53], the severest form of visceral disease. Patients mainly present with symptoms of radiculomyelitis or radiculomyeloencephalitis, eosinophilic meningitis or meningoencephalitis, subarachnoid and even intracerebral hemorrhage [54–56]. Human neurognathostomiasis has a long history, with the first case reported in 1949 and the first pathologic evidence documented in 1967 [57, 58]. Since then, the detection of neurognathostomiasis has increased steadily due to improved diagnostic techniques.

*Gnathostoma* L_3_ are highly invasive, and migrate by releasing various molecules, such as cysteine proteases and matrix metalloproteinases (MMPs) into the invaded micro-environment to promote their penetration and invasion of organs [59, 60]. Larvae invade the CNS directly through the loose connective tissues of the neural foramina of the skull base, and the intervertebral foramina of the spine and vessels [61]. Radiculomyelitis can be caused by larvae entering the nerve roots of the spinal cord [62]. Migration of the larvae within the CNS can also cause mechanical injury, parenchymal damage and subarachnoid hemorrhage [63]. The salient symptoms and signs of neurognathostomiasis are the sudden onset of severe radicular pain with headache followed by loss of function of the cranial nerves and paralysis of the extremities, or quadriparesis with bladder dysfunction; the initial pain is characteristically followed by degrees of paralysis, ranging from weakness to thorough paralysis of one to all four limbs [56, 64]. Further migration of the larvae within the CNS may lead to a multiplicity of rapidly progressing lesions beyond the extent of cerebral edema [1].

Direct mechanical damage to the CNS can also occur, as the relatively large L_3_, averaging 3–4 mm in length, can migrate through neural or vascular tissue [65]. Larvae burrowing through a cerebral arteriole may cause subarachnoid hemorrhage. The universal presence of eosinophilic pleocytosis indicates that inflammatory responses to larval invasion could lead to further tissue destruction [64].

Should migrating larvae invade vital structures in the brain stem, death can occur after several days following the onset of symptoms [56]. High-resolution magnetic resonance imaging (MRI) can be used to image the tracks of *Gnathostoma* L_3_, and greatly increases the accuracy of diagnosis of neurognathostomiasis [66].

## Epidemiology

**Fig. 2:**
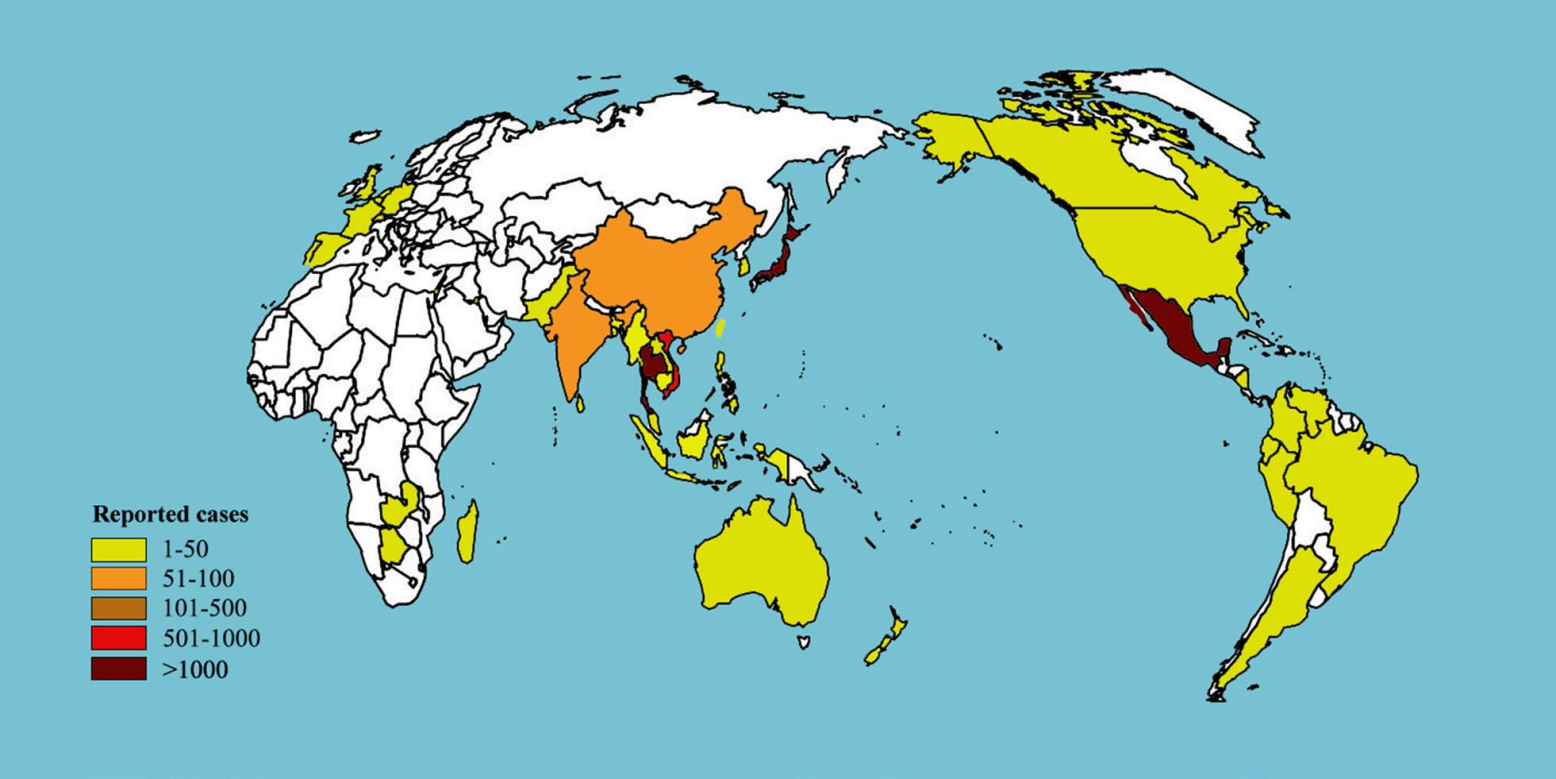
Map of countries with reported cases of gnathostomiasis

*Gnathostoma* spp. have a worldwide geographical distribution. About 5000 cases of human gnathostomiasis have been reported worldwide since the first one was described in Thailand in 1889. Gnathostomiasis is endemic in Japan and Thailand [1], and has been sporadically reported in numerous countries around the world (Fig. 2).

Five *Gnathostoma* species have been found to infect humans. *G. spinigerum* is commonly found in China, India, Japan and Southeast Asia; *G. hispidum* is found in Asia, Australia and Europe; *G. doloresi* is found in Southeast Asia; *G. nipponicum* is distributed in Korea and Japan [1]; and *G. binucleatum* is found in Mexico and some South American countries.

In Japan, 3182 cases of human gnathostomiasis were reported from 1911 to 1995, with 103 cases in which a *Gnathostoma* worm was detected [22, 54, 67–74]. Seventy three cases were reported from 1996 to 2012, with the detection of L_3_ in 29 cases (personal communication, unpublished data). In Thailand, there have been 1079 recorded cases of human gnathostomiasis. The seroprevalence of *Gnathostoma* in humans was 62.5% (531/849) in Bangkok, Thailand between 2000 and 2005 [75]. The high prevalence of gnathostomiasis in this population might be partly due to the local custom of eating raw fish [75]. In China, the first case of human gnathostomiasis was reported in Xiamen, Fujian province in 1919. Eighty-six cases (83 of which were caused by *G. spinigerum*, two by *G. hispidum* and one by *G. doloresi*) were reported between 1918 and 2014, mostly in southern and eastern China [41, 67, 68, 76–80]. Among these cases of human gnathostomiasis, more than 90% were due to the ingestion of raw or undercooked food (mostly fish, including eels and loach, but also frogs and snakes) [81].

## Social-ecological status

Although the presence of intermediate hosts is necessary for the endemism of gnathostomiasis, dietary habits are a key factor in its transmission. As mentioned earlier, eels, loaches, frogs and snakes, considered delicacies by some ethnic populations, are the most important second intermediate hosts of *Gnathostoma* spp. [1]. An increasing number of people can afford these delicacies due to the improvement of living standards. Freshwater fish (including eels and loaches), either raw or marinated in lemon juice, such as in sushi, sashimi and ceviche, are very popular food items worldwide [82, 83]. In many countries and regions, offering raw fish to guests is deemed a hospitable gesture. Many people mistakenly believe that raw fish are highly nutritious and that the L_3_, if present within them, can be killed by the concurrent consumption of alcohol or hot spices. It is also known that smoking or pickling may not always be effective in killing L_3_ [1]. Adequate cooking is the most effective way of ensuring that the larvae are killed, although freezing infected food at −20 °C for 3–5 days is also effective [1].

A high demand for exotic foods such as eel, loach, frog and snake has led to the rapid expansion of aquaculture around the world, and rivers, lakes and water reservoirs are now widely used to increase their cultivation [18]. Pigs are definitive hosts of *Gnathostoma* spp. Many small pig farms in developing countries are purposely built so that the swine feces end up in a pond/river/lake as feed for aquatic animals. However, pigs may be infected with *Gnathostoma* spp., in which case the eggs in their feces can act as the source of infection of intermediate hosts [84].

## Diagnosis

The diagnosis of human gnathostomiasis is based on clinical symptoms and signs (intermittent subcutaneous or cutaneous migratory swelling), an elevated blood eosinophil level and a relevant exposure history (living in or traveling to endemic regions; ingesting raw or undercooked fish, frog or chicken) [85]. Subcutaneous gnathostomiasis commonly presents as a single nodule; in contrast, multiple nodules often exist in other parasitic infections such as sparganosis and cysticercosis [86]. A final diagnosis of gnathostomiasis can be established upon surgical removal of L_3_ or identification of the worms in a tissue specimen along with eosinophilia [8, 46, 87]. The accurate identification and differentiation of various *Gnathostoma* species have traditionally been based on morphological features [88]. However, the genus *Gnathostoma* includes 12 different species, five of which infect humans, that are virtually indistinguishable based on morphology, particularly at the larval and/or egg stages, which raises questions about *Gnathostoma* taxonomy [89].

Molecular techniques provide a definitive alternative approach to morphological identification and differentiation of *Gnathostoma* species. PCR-based approaches such as amplicon sequencing are a rapid and sensitive means of identification, and can be used for the phylogenetic analysis of different *Gnathostoma* species. The most commonly targeted genetic markers, namely nuclear small subunit ribosomal RNA (rRNA), internal transcribed spacer (ITS) regions of nuclear ribosomal DNA (rDNA) and the mitochondrial (mt) cytochrome c oxidase subunit 1 (*cox1*) gene, have been used to study genetic variation in *Gnathostoma* [90–93]. PCR-coupled sequencing and bioinformatics methods have also been used to identify and differentiate *Gnathostoma* in fixed and paraffin-embedded tissues, and can be used for the reappraisal of individual cases [94].

Gnathostomiasis can also be diagnosed using antigen-specific immunoglobulin G (IgG) antibodies, although the detection of *Gnathostoma* spp. larvae is the gold standard for its diagnosis. An enzyme-linked immunosorbent assay (ELISA) for L_3_ IgG antibodies has been developed. However, its sensitivity and specificity have been shown to be poor, i.e. 59–87% and 79–96%, respectively [95]. Some studies reported a significant improvement in the diagnosis of human gnathostomiasis, although the IgG_2_ antibody showed cross-reactivity with several other nematode species [96]. Currently, an inability to accurately identify the infecting species of *Gnathostoma* is a major limitation in the diagnosis of human gnathostomiasis. Serological tests often show limited species identification due to antigenic cross-reactivity between species. A recent study indicated that recombinant MMPs of *G. spinigerum* can be used for the serodiagnosis of neurognathostomiasis [97].

Neuroimaging is non-specific and non-confirmatory, but can be used to complement serological tests to provide a presumptive diagnosis of human gnathostomiasis. Medical imaging techniques such as CT, MRI and ultrasonography can be used to assist the clinical diagnosis of patients with visceral disease [98–100]. MRI is superior to CT in the neuroimaging of cerebral larva migrans caused by *Gnathostoma* spp. A presumptive diagnosis of gnathostomiasis in cases where larvae have not been recovered can be reached by a combination of positive neuroimaging and immunoblot [60]. However, accurate diagnosis by imaging heavily depends upon infection intensity. Immunochromatographic test kits are promising diagnostic tools for rapid clinical diagnosis at the site of care and also for epidemiological surveys [101].

## Treatment and control

There is no effective non-invasive treatment for human gnathostomiasis, and the surgical removal of larvae is considered the most effective treatment for this disease [1]. However, surgical removal is only feasible in cases of cutaneous or other types of superficial migration. For most cases of visceral gnathostomiasis, surgical removal is impracticable if not impossible. In these cases, various drugs (thiabendazole, praziquantel, metronidazole, diethylcarbamazine, and quinine) have been tested, but have shown no obvious efficacy [102].

Albendazole is the drug of first choice for human gnathostomiasis. A recommended dose of 400 mg twice a day for 21 days resulted in a cure rate of > 90% [103]. Ivermectin has similar therapeutic efficacy to that reported for albendazole [104], and has been shown to be effective at either 0.2 mg/kg as a single dose or at 0.1 mg/kg administered on 2 consecutive days. Corticosteroids may be administered alone (prednisolone, 60 mg/day for 7 days), and cause the larvae to migrate and then die naturally [51]. Nevertheless, steroids should be used with caution in cases of ocular or CNS gnathostomiasis due to their potential to cause further larval migration.

Initial chemotherapy is usually unsuccessful, as the majority of patients relapse and then require a second or even a third course of albendazole or ivermectin therapy. Relapses are often heralded by the appearance of peripheral eosinophilia [105]. However, in a few cases, albendazole or ivermectin has been used as an initial treatment with successful outcomes. A recent report by Gui et al. [106] showed that albendazole at 400 mg/day for 10 days successfully cured two patients with pulmonary gnathostomiasis.

## New insights into human gnathostomiasis from the “omics” sciences

The complete elucidation of four *Gnathostoma* mt genomes was an important step towards a better understanding of these parasites, and the disease that they cause, at the molecular level. Genomic data could prove useful for the reassessment of phylogenetic relationships, and for the development of next-generation diagnostics and therapeutic interventions. Complete mt genomes have been elucidated for *G. spinigerum* (14,079 bp) [107], *Gnathostoma* sp. (14,391 bp), *G. nipponicum* (14,093 bp) [108], and *G. doloresi* from China (13,809 bp) and Japan (13,812 bp) [109]. These mt genomes encompass 36 genes including two rRNA genes, 22 transfer RNA genes and 12 protein-coding genes with the *atp8* gene missing [107–109]. The inference of amino acid sequences from mt genome sequences is necessary for the systematic analysis of the relationships between *Gnathostoma* and other nematodes at the molecular level. Concatenated mt proteomic datasets have been shown to be very useful for re-examining the systematic relationships of different nematode groups [110–115]. Because of the strong phylogenetic signals and statistical support in phylogenetic trees generated from mt proteomic datasets of members of the suborder Spirurina [34, 116], it is now considered timely to examine the phylogenetic relationships of many spirurine nematodes. Molecular tools that use genetic markers such as rDNA ITS sequences and mt *cox*1 have been examined for their application to the clinical diagnosis of *Gnathostoma* infection [91, 93]. Sequence heterogeneity in ITS rDNA can be high in individual nematode specimens [117], and the protein-coding genes of the mt genome are reasonably predicted to be better suited for this type of analysis [118]. This can be achieved by using PCR-coupled single-strand conformation polymorphism and DNA sequencing [119]. This technique has already been applied, on a small scale, to *G. spinigerum* [92]. A comparative study of DNA sequences indicated that the mt *cox1* gene can be used as a genetic marker for the identification and differentiation of *Gnathostoma* species [93]. Mt *cox1* sequences showed a relatively high degree of genetic variability (four distinct haplotypes) among *G. spinigerum* specimens from different host species (i.e. dogs, snakes and eels) and localities within Asia and Southeast Asia (i.e. China, Indonesia, Laos and Thailand) [120]. An assessment of the various haplotypes or genotypes of *Gnathostoma* and how they relate to different clinical signs of gnathostomiasis in humans would be useful.

Proteomic analyses of *G. spinigerum* are increasingly recognized for their value in the study of parasite biology and host-parasite interactions. Various biological and pathological functions of antigenic proteins of *G. spinigerum* have been identified, which include responses to stress, metabolic processes and energy generation, proteolysis, cell skeleton formation, protein folding, oxidation-reduction and carbohydrate ligand binding [121]. Immunoproteomic analysis has identified a number of antigenic proteins of *G. spinigerum* with potential as vaccine candidates for *G. spinigerum* infection.

Genome, developmental transcriptome and microRNA datasets constitute a collective resource for future investigations into the molecular biology, immunobiology, phylogenetics, epidemiology, population genetics and pathogenesis of *Gnathostoma* and/or gnathostomiasis. They should also be useful for the improvement of diagnostics and development of new drugs, including anthelmintics and vaccines [122]. Future studies should focus on: (i) sequencing and annotating the genome of *G. spinigerum*, and comparing it with those of other nematodes, with particular emphasis on excretory and secretory protein-encoding genes that are predicted to be involved in host invasion and parasite-host interactions; (ii) developmental transcriptome or microRNA datasets, which may prove useful for a better understanding of the biology and physiology of *Gnathostoma* nematodes.

## Conclusions and future directions

While soil-transmitted helminths have received much attention because of their major socioeconomic impacts [123], other types of helminths such as *Gnathostoma* spp. have been largely neglected. Despite the fact that epidemiological studies of gnathostomiasis have been reported from many countries worldwide, gaps exist in our understanding of the epidemiology of *Gnathostoma* infection, and its zoonotic importance remains ill-defined. Combined with the lack of any estimate of the global burden of gnathostomiasis, all of these factors serve to limit a proper assessment of the public health impact and burden of gnathostomiasis.

Deciphering the genomes of *Gnathostoma* and their transcription will assist investigations into the immunobiology of this genus, as well as provide a genetic basis for the epidemiological study of these parasites. This will also facilitate studies on the biology, biochemistry, and physiology of these parasites, and the molecular mechanisms involved in their ability to modulate or evade the host immune system [124–126]. MicroRNAs have been assessed for their diagnostic value in several nematode infections [127–129], and the identification of specific biomarkers for the diagnosis of gnathostomiasis is a promising direction for future investigations.

The public health importance of helminthic infections, including gnathostomiasis, has been seriously neglected worldwide. The devastating consequences of this include the persistence and ever-increasing number of cases of gnathostomiasis, and the corresponding heavy disease burden. Human gnathostomiasis is now considered an important food-borne parasitic zoonosis [15, 130]. Human beings are infected with *Gnathostoma* spp. mainly by consuming raw or undercooked food (fish, frogs, eel, poultry and snakes) that contains the parasite larvae. Dogs, cats, snakes, fish and birds all play important roles in the transmission of this disease. Recommended measures for the prevention and control of human gnathostomiasis primarily focus on educational campaigns in an effort to change eating habits. First, adequate cooking of potentially infected food is the safest way to ensure that larvae are killed, thereby preventing infection. Secondly, treating the definitive hosts, such as dogs and cats, with anthelmintics minimizes the source of infection. Thirdly, public awareness needs to be increased to promote a reduction in the hunting and sale of wildlife—especially wild birds, loaches, eels and snakes—for human consumption.

## Data Availability

All datasets supporting the conclusions of this article are included within the article.

## Abbreviations

AdL_3_: Advanced third-stage larvae
CNS: Central nervous system
*cox1*: Cytochrome c oxidase subunit 1
CT: Chromatographic test
ELISA: Enzyme-linked immunosorbent assay
EL_3_: Early third-stage larvae
ES: Excretory–secretory
IgG: Immunoglobulin G
ITS: Internal transcribed spacer
L_1_: First-stage larvae
L_2_: Second-stage larvae
L_3_: Third-stage larvae
MMPs: Matrix metalloproteinases
MRI: Magnetic resonance imaging
mt: Mitochondrial
PCR: Polymerase chain reaction
rDNA: Ribosomal DNA
rRNA: Ribosomal RNA
